# Chemical Feedback in Templated Reaction-Assembly of Polyelectrolyte Complex Micelles: A Molecular Simulation Study of the Kinetics and Clustering

**DOI:** 10.3390/polym15143024

**Published:** 2023-07-12

**Authors:** Christos Gioldasis, Apostolos Gkamas, Othonas A. Moultos, Costas Hristos Vlahos

**Affiliations:** 1 Chemistry Department, University of Ioannina, 45110 Ioannina, Greece; c.gioldasis@uoi.gr; 2 Engineering Thermodynamics, Process & Energy Department, Faculty of Mechanical, Maritime and Materials Engineering, Delft University of Technology, Leeghwaterstraat 39, 2628 CB Delft, The Netherlands; o.moultos@tudelft.nl

**Keywords:** molecular dynamics, polyelectrolytes, polymerization, self-assembly, graph theory

## Abstract

The chemical feedback between building blocks in templated polymerization of diblock copolymers and their consecutive micellization was studied for the first time by means of coarse-grained molecular dynamics simulations. Using a stochastic polymerization model, we were able to reproduce the experimental findings on the effect of chemical feedback on the polymerization rates at low and high solution concentrations. The size and shape of micelles were computed using a newly developed software in Python conjugated with graph theory. In full agreement with the experiments, our simulations revealed that micelles formed by the templated micellization are more spherical and have a lower radius of gyration than those formed by the traditional two-step micellization method. The advantage of molecular simulation over the traditional kinetic models is that with the simulation, one studies in detail the heterogeneous polymerization in the presence of the oppositely charged template while also accounting for the incompatibility between reacted species, which significantly influences the reaction process.

## 1. Introduction

Polyelectrolyte (PE) complex micelles (PCM), which are formed in aqueous solutions by mixing charged ionic-neutral double hydrophilic diblock copolymers with oppositely charged homopolymers, have recently attracted much attention [1,2,3,4,5,6] due to their potential use as nanocarriers and nanoreactors [7,8]. The oppositely charged moieties in the mixture form the PCM core, since the solvent conditions for these moieties worsen due to charge neutralization [9]. The PCM corona is formed of the neutral hydrophilic block. Although the existence of opposite charges on the PE moieties is the necessary condition for electrostatic complexation to occur, the contribution of electrostatics on the Gibbs free energy of mixing becomes significant only at high ionic strengths [10]. In sharp contrast, at low ionic strengths, the driving force of complexation is the entropy gain from the release of a large number of small counterions in the solution [10].

**Figure 1:**
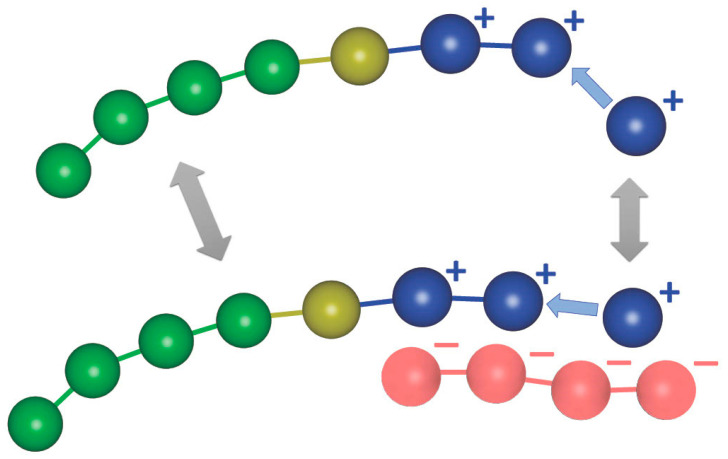
Cartoon representation of the coupled reactions in templated polymerization. Single arrows indicate the monomer polymerization. Double arrows indicate the exchange of monomers and diblock copolymer chains between the two reactions (chemical feedback). Color code: neutral block beads (green), initiator beads (yellow), negatively charged template beads (red), and positively charged monomers (blue).

PCMs can be experimentally synthesized in two different ways. The most common is by using pre-synthesized building blocks and subsequent co-assembly to form PCMs. The other synthetic pathway is the polymerization-induced electrostatic self-assembly (PIESA). In PIESA [11,12], the polymerization of the charged polyelectrolyte block of the copolymer is templated by the oppositely charged homopolymer chain with simultaneous co-assembly in a one-pot reaction. The spatial and time colocalization of covalent and supramolecular electrostatic assembly, involving the same molecular compounds, results in chemical feedback between the different primary reactions, i.e., the polymerization in the solution and the polymerization on the template (Figure 1). Chemical feedback in the coupled reactions-assembly has profound effects on both the kinetics of polymerization and the final size and shape of PCMs.

Bos et al. [13] studied the effects of chemical feedback on the kinetics of the reversible addition-fragmentation chain-transfer (RAFT) polymerization of positively charged monomers of vinylbenzyltrimethylammonium cloride (VBTAC) with a PEG chain-transfer agent containing 220 monomers and poly(sulfopropylmethacrylate) (PSPMA) as the negative template with a degree of polymerization (DP) equal to 47. The target length of the VBTAC block in the (PEG)-b-(VBTAC) copolymer was 50, with a monomer to template sites ratio of 1:1. The authors showed that the polymerization rate is strongly enhanced when the template is used at a solution concentration [*Φ*] ≈ 0.03. This is because the binding of the charged monomer to the template increases the local monomer concentration near the template compared to the monomer concentration in the solution in the non-templated polymerization. The increase in [*Φ*] to 0.09 was shown to not further affect the polymerization rate. In the same study, the average micelle size in the templated polymerization was found to be smaller than the size of micelles formed by the addition of the template after the polymerization of the charged block of the copolymer. 

In a similar experimental study, Ding et al. [14] reported the exact opposite results of Bos et al., but at a higher solution concentration ([*Φ*] ≈ 0.16 and 0.5). Ding et al. performed RAFT polymerization of 2-acrylamydo-2-methylpropanesulfonic (AMPS) acid monomers with targeted DP = 50–150. Poly(2-hydroxypropyl methacrylamide) (PHPMA) was acting as CTA and polyethylimine (PEI) as a template with DP = 232 and 44, respectively. In that study, the templated polymerization proceeded slower than the homogeneous solution polymerization. The kinetic model of Bos et al. [13] was unable to describe the experimental findings of Ding et al. It was hypothesized that the 17 times larger template concentration used in experiments by Ding et al. causes an increase in the viscosity, and thus, slows down the overall polymerization kinetics.

Kinetic models are based on the numerical solution of kinetic equations in the approximation of an absolutely homogeneous system, i.e., instant balancing of all concentrations of all components in space and time [15]. In contrast, molecular simulations can be used to study in detail the heterogeneous polymerization in the presence of the oppositely charged template and also to account for the incompatibility between reacted species that significantly influences the reaction process. Simulations of RAFT polymerization accounting for the main reactions of the experimental process in the synthesis of linear homopolymers were performed by Gavrilov et al. [15] Using a Monte Carlo algorithm implemented with dissipative particle dynamics, they found that if the RAFT/initiator ratio is large, a simplified model with no termination and intermediate radical formation can be used with good enough accuracy. Using the simplified model, they studied the heterogeneous polymerization-induced self-assembly (PISA). It was shown that the incompatibility between species results in different chain length distributions and polydispersity. The latter noticeably changes the phase behavior of the copolymer and the micelle size.

Due to the lack of similar experiments studying a wide range of concentrations, molecular simulation can be used to understand the underlying effects of chemical feedback on the kinetics and the size of the resulting PCMs. To this purpose, we performed coarse-grained molecular dynamics (CGMD) simulations using a stochastic reaction model (SRM) for the polymerization of the charged copolymer block. This approach has been successfully used to study radical and living polymerizations in solution, bulk, and flat surfaces [16,17]. In addition, CGMD has been widely used to study the self-assembly behavior of copolymers [18,19,20]. Here, we computed the polymerization rate constants, the local monomer concentrations, and the polydispersity of the synthesized diblock copolymer for both the templated and non-templated polymerization to explain the experimental findings. The variation of these properties with the total solution concentration, the neutral block length, the targeted polymerization length, the template length, and the excluded volume interactions between the template and the monomers were also calculated. A new clustering algorithm in Python based on graph theory was developed to compute the size and shape of micelles obtained from polydisperse diblock copolymers for the templated PIESA and the non-templated polymerization followed by the assembly with the addition of the template.

## 2. Model

### 2.1. Coarse-Grained Molecular Dynamics Simulation Details

All CGMD simulations were performed using the open-source Large-scale Atomic/Molecular Massive Parallel Simulator (LAMMPS [21]). Periodic boundary conditions were imposed in all directions. The Murat–Grest bead–spring model [22,23,24,25] was used to describe homopolymer chains consisting of neutral beads (A type), templates of negatively charged beads (C type), positively charged monomer beads (B type), initiators (I), and counterions. The van der Waals and electrostatic interactions were modeled by the Lennard–Jones (LJ) and Coulombic potentials, respectively. The bonded interactions were modeled using the finitely extensible nonlinear elastic (FENE) potential [22]. The simulations were performed using dimensionless units. A bond stiffness (*k*) of 25*ε*/σ^2^ (where *ε* and σ are the LJ parameters, both set equal to 1) and a maximum bond extension distance (*r*) of 1.5σ were used. The solvent was implicitly treated via the Langevin thermostat [20]. The long-range electrostatic interactions between the charged beads were handled using the particle–particle particle–mesh (PPPM) method [26] with Bjerrum length *l*_B_ = 1. The real-space cutoff was set to 5.0σ. Different cut-off distances in the LJ potential were used [9,20] to describe the interactions between beads. C–C and monomer-monomer interactions were considered attractive with *r*_cij_ = 2.5σ. All the other interactions were considered repulsive with *r*_cij_ = 2^1/6^σ.

The simulations were performed at a reduced temperature (T* = *k_B_T*/*ε* = 2), corresponding to bad solvent conditions [22] for C–C and monomer–monomer interactions. The solvent conditions for the charged moieties are determined from the balance of hydrophobic attractions between beads and the electrostatic repulsions between charges; for non-neutralized charges, the electrostatic repulsions are predominant; therefore, charged moieties are hydrophilic. Conversely, the neutralization of charges leads to the predominance of attractions between beads, making neutralized moieties hydrophobic. All types of beads were considered to have the same mass (m = 1).

In the simulations of the non-templated polymerization, mixtures containing 500 homopolymer chains and positively charged monomers and counterions were used. The homopolymers consist of 25, 50, and 100 neutral A-type beads (i.e., A_25_, A_50_, A_100_). The number of monomers and counterions is determined by the target length of the polymerized copolymer block consisting of 20 type beads (B_20_). In all simulations, 1500 I-type beads were used. In the templated polymerization simulations, negatively charged templates consisting of 20, 40, 80, and 125 C-type beads (C_20_, C_40_, C_80_, C_125_) were added to the mixtures. These are shown as red chains in Figure 1. The ratio of charged monomers to oppositely charged template beads was set equal to 1:1 in most of the simulations since it was verified [9,13] that this leads to a high number of micelles with reasonable aggregation numbers (up to *N* = 150). Simulations with a ratio of 1:2 were also performed to compare with experimental findings [13]. All solutions are electroneutral with the addition of the appropriate number of counterions. The total concentration of beads of all types in the simulation box was varied according to [*Φ*] = 0.04, 0.12, 0.24, and 0.36. 

In all simulations, initially, 1 million time steps were performed with an integration step of $\Delta t=0.006\tau \;(where\;\tau =\sqrt{\frac{m\sigma^{2}}{\varepsilon }})$, setting all cutoff radii equal to r_cij_ = 2^1/6^σ to eliminate any biases introduced from the initial conformation. Then, the systems were allowed to equilibrate for half a million time steps. 

### 2.2. Modeling the Polymerization 

**Figure 2:**
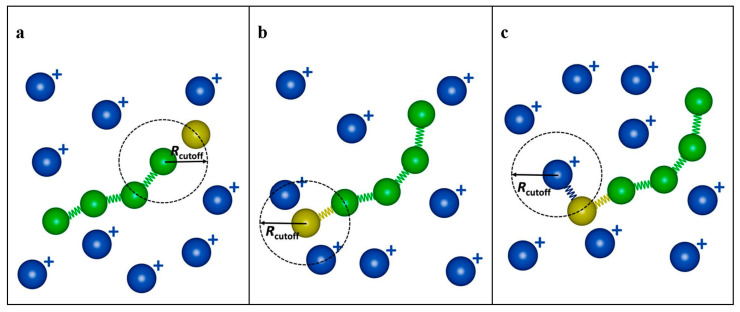
Cartoon representation of Monte Carlo “bond/create” algorithm (**a**) cutoff distance for bond/create algorithm, (**b**) activation of a neutral chain end by the addition of the initiator, and (**c**) the bond creation between the homopolymer active center and the charged monomer.

For the stochastic polymerization of the positively charged monomers for the synthesis of linear AB diblock copolymers shown in Figure 2, the “bond/create” functionality of LAMMPS was used. This functionality is based on a Monte Carlo algorithm that creates new bonds between atoms according to specific criteria. Possible bond pairs are identified when two non-bonded beads (*i* and *j*) are within a set distance (R_cutoff_) of each other, given that the maximum number of bonds allowed per bead is not reached (i.e., 2 for linear chains). If multiple neighbors are within the R_cutoff_ of a bead, the closest one is chosen as the sole bond partner. This bond can be created based on a predetermined reaction probability (RP). The number of maximum bonds and the types of beads can be changed after a successful bond creation. A check for possible new bonds is performed every *N*_every_ time step during the simulation. A schematic representation of the “bond/create” scheme is shown in Figure 2.

Three different polymerization steps were performed here: (a) the activation of the neutral homopolymer chain end bead by the addition of initiator beads (Figure 2b), (b) the bond creation between the homopolymer active center and the monomer (Figure 2c), and (c) the propagation of polymerization for the synthesis of the charged B block of the diblock copolymers. The addition of the neutral initiator alters the length of the neutral block by one bead, e.g., A_50_ to A_51_. Since the “bond/create” is a Monte Carlo algorithm, only one of the polymerization steps can be executed at a time. This means that only one bond can be created per time step. *R*_cutoff_ was set equal to 1σ in all simulations. This stochastic algorithm approaches the achievements of RAFT polymerization in the sense that the main target of RAFT polymerization is to find the suitable stoichiometry (and the appropriate probabilities for the different steps in the simulation) in order for the polymerization of the diblock copolymer block to take place in the vast majority from the end of the neutral block [15]. Our algorithm allows all monomers to polymerize exclusively from the end of the neutral block. As we mentioned in the introduction, if the RAFT/initiator ratio is large, a simplified model with no termination and intermediate radical formation, as in our simulations, can be used with good enough accuracy [15]. Thus, our model is a coarse-grained model of the full RAFT mechanism.

### 2.3. Clustering Analysis

In the templated reaction assembly, after the polymerization phase is complete, the simulation was carried out for 60 million time steps with integration step Δt=0.006τ. The duration of the simulation was determined by the relaxation time of the tracer autocorrelation function [27,28] (Equation S1 in the Supporting Information) of the instantaneous chains involved in a micelle. In the non-templated reaction, after the completion of the polymerization, simulations with the newly formed AB diblock copolymers along with template chains and counterions are performed to study the micellization at the desired concentrations. These simulations were performed for 15 million time steps with Δt=0.006τ. The properties of interest are calculated from 2000 to 4000 snapshots using the block average method with ten blocks. Following the Stillinger criterion [29], a diblock copolymer and a template chain were assumed to reside in the same micelle if any two oppositely charged beads (B and C) were found within 1.5σ. In our previous study [9] on the micellization through complexation of oppositely charged diblock copolymers, we have shown that two beads of identical charge to be within 1.5σ is highly unlike. Thus, clustering algorithms that do not distinguish the charge types of the beads can safely be used for the micellization study of oppositely charged polymers.

We used graph clustering analysis to analyze the simulation data. We first identified the micelles with the data clustering algorithm DBSCAN implemented in the Python library Sklearn [30] with a maximum allowable neighborhood radius of 1.5σ. For a point (bead) to be considered a core point, at least two points (including the point itself) must be in the neighborhood. We used a precomputed neighbor sparse array as the input to the DBSCAN algorithm. To compile this array, we used the KDTree neighbor data structure from the Python library SciPy [31], in particular, the Sparse Distance Matrix algorithm with the max distance between two points of 1.5σ (note that the distance matrix algorithm ignores points with a distance greater than the max distance parameter). The KDTree neighbor data structure takes periodic boundary conditions into account, and thus, the clustering analysis includes the periodic images. 

To identify polymer chains, we used the Python NetworkX library [32]. Accordingly, beads were represented as nodes, and bonds were represented as edges. From the graph created by this library, we could extract the polymer chains using the algorithm “Connected/Components”. This algorithm generates connected components from a graph, i.e., bead spring chains in our case. Then, the polymer chains were assigned to micelles based on the previous steps. To compute properties such as the radius of gyration of the micelles (core, corona, and total) and the shape anisotropy parameter *κ*^2^ (Equation S2 in the Supporting Information) the outbox coordinates were used. To this purpose, micelles split due to the periodic conditions (inbox coordinates) were determined and unified using the data clustering algorithm DBSCAN, KDTree neighbor data structure, and Sparse Distance Matrix algorithm without periodic conditions. 

## 3. Results and Discussion

### 3.1. Polymerization Rate

**Figure 3:**
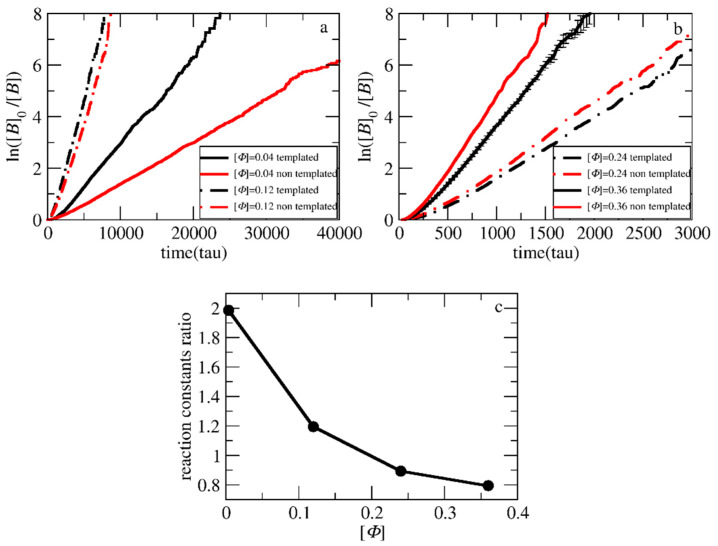
Pseudo-first-order kinetic plot of templated and non-templated polymerization for the synthesis of A_51_B_20_ diblock copolymers at total solution concentrations of (**a**) [*Φ*] = 0.04, 0.12 and (**b**) [*Φ*] = 0.24, 0.36. (**c**) The ratio of reaction constants of the templated to non-templated polymerization at different [*Φ*]. The template is a C_20_ chain. Error bars represent standard deviation.

The kinetics of polymerization of the cationic monomers for the synthesis of AB diblock copolymers can be described by a pseudo-first-order reaction [13] according to

(1) $$ln\left(\frac{{\left[B\right]}_{0}}{\left[B\right]}\right)=kt$$

where [*B*]_0_ is the initial monomer concentration, [*B*] is the monomer concentration, *k* is the polymerization rate constant, and *t* is the time. To determine the effect of chemical feedback on the polymerization rate in the templated reaction when the concentration of monomers, the length of the neutral block, and other parameters vary, we compared it with the respective polymerization rate of the non-templated polymerization, in which there is no assembly, and hence, no chemical feedback. To investigate the influence of concentration on chemical feedback, simulations of mixtures containing 500 neutral homopolymer chains A_50_, 10,000 positively charged B-type monomers, 1500 initiator beads, and 10,000 counterions were performed. For the templated polymerization, 500 additional templates C_20_ and 10,000 counterions were considered. The reaction probability was set to 0.125. Using different simulation box sizes, concentrations of [*Φ*] = 0.04, 0.12, 0.24, and 0.36 were achieved. The target length of the polymerized positively charged block of the copolymer was set to 20 beads (A_51_B_20_). The kinetic plots obtained from the simulation are illustrated in Figure 3. The resulting polymerization rate constants are presented in Table S2.

**Figure 4:**
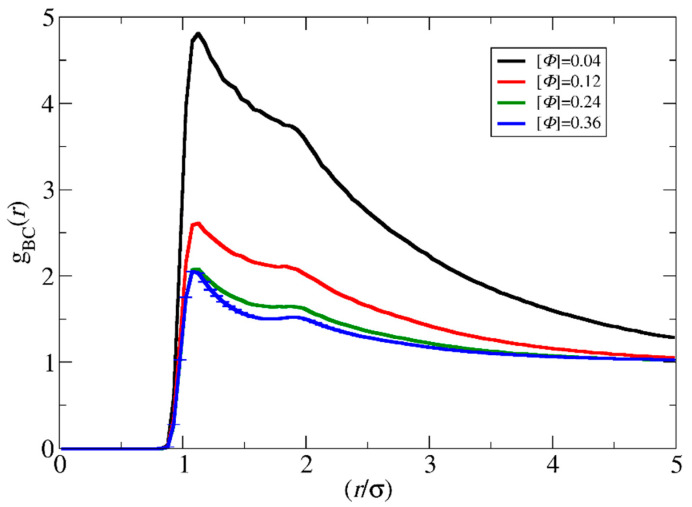
The radial distribution function *g*_BC_(*r*) between the template C and monomer B at different solution concentrations ([*Φ*]) for the synthesis of A_51_B_20_ diblock copolymers. The template is a C_20_ chain. Error bars represent standard deviation.

As can be observed, the polymerization rate in the templated reaction at [*Φ*] = 0.04 is much faster than in the non-templated polymerization (Figure 3a,c). This is in full agreement with the experimental results of Bos et al. [13] reported for similar concentrations. The increase to [*Φ*] = 0.12 increases both the reaction rates. However, the ratio of the rates of the two polymerization types becomes much smaller. Further increase to [*Φ*] = 0.24 and 0.36 (Figure 3b,c) leads to the opposite result, i.e., the non-templated polymerization rate becomes higher than the templated polymerization. This is in full agreement with the experimental results by Ding et al. [14] for concentrations of 16% and 50% *w*/*w* (coacervate in water). Since the reaction probability in the simulations is constant in both cases, the local monomer concentration before the polymerization takes place may be a clear measure of the complex dependency of the polymerization rate on the concentration. To quantify the local monomer concentration in the templated polymerization, the radial distribution functions *g*(*r*_CB_) of the template and the monomer beads were calculated from two hundred simulation snapshots obtained by a trajectory of one million time steps after the initial equilibration (Figure 4). Three independent simulations of the mixture with [*Φ*] = 0.36, each one starting from a different initial configuration, were used for the calculation of the standard deviation of *g*(*r*_CB_). The integration of *g*(*r*_CB_) up to a radius of ca. 3.5σ yields the number of B-type beads that surround each C-type bead. From this number, the local concentration of monomers within the spherical volume with this radius can be computed. For the non-templated polymerization, the calculation of the local concentration of monomers is straightforward because the system is homogenous without template beads and is equal to the total monomer density in the simulation box.

**Figure 5:**
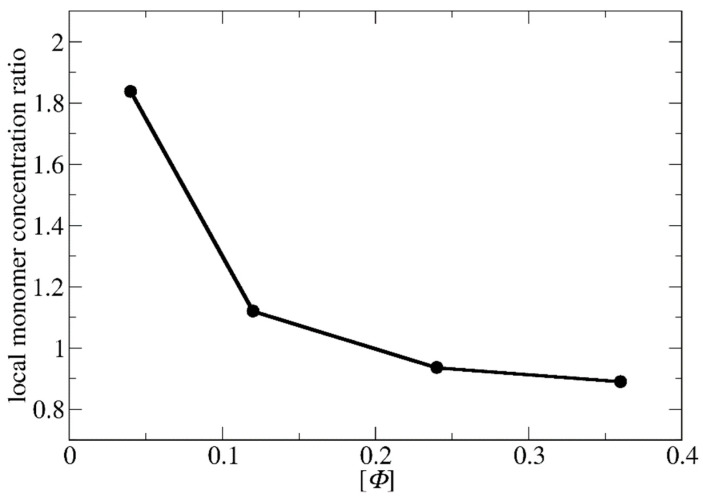
The variation of the ratio of the local monomer concentration of the templated to non-templated polymerization with the total solution concentration ([*Φ*]) for the synthesis of A_51_B_20_ copolymers. The template is a C_20_ chain.

As shown in Figure 5 and Table S2, the ratio of local monomer concentrations of the templated to the non-templated polymerizations decreases with the total concentration in a similar way as the ratio of polymerization rate constants decreases with [*Φ*] (Figure 3c). This shows that the local concentration may be the key parameter for understanding the variation of polymerization rate with the concentration. At [*Φ*] = 0.04, the local concentration of the monomers around the template is two times higher than the respective of the non-templated solution, accelerating this way the templated polymerization. The difference in local monomer concentrations for the two types of polymerizations decreases at [*Φ*] = 0.12. Nevertheless, the local monomer density in the templated polymerization is always higher, leading to a higher polymerization rate. At the most concentrated systems ([*Φ*] = 0.24 and 0.36), the LJ interactions have a strong impact on the templated polymerization rate. The repulsion between the template and the monomers prevents them from coming close, and thus, both the local density and the polymerization rate in the templated reaction become smaller than in the non-templated reaction.

**Figure 6:**
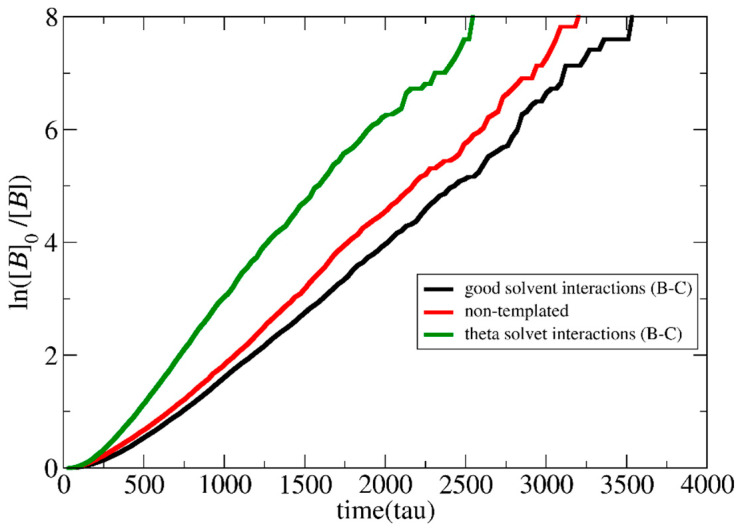
Pseudo-first-order kinetic plot of templated and non-templated reaction for synthesis of A_51_B_20_ diblock copolymers for good and theta solvent interactions between templated C and monomer B type beads. The total solution concentration is [*Φ*] = 0.24. The template is a C_20_ chain.

To study the effect of LJ interactions, separate simulations with the LJ interaction parameters set to *ε*_BC_ = 1, *ε*_BB_ = 1.5, and *ε*_CC_ = 1.5 at T* = 3.0 were performed at [*Φ*] = 0.24. In this way, the B–B and C–C type interactions remain in bad solvent conditions as previously, but the B–C interactions correspond to theta solvent for the neutral chains [9]. As shown in Figure 6, the templated polymerization rate becomes much higher than in the non-templated case since both the neutralized template and the monomers prefer to be close to each other, increasing the local concentration around the template. 

**Figure 7:**
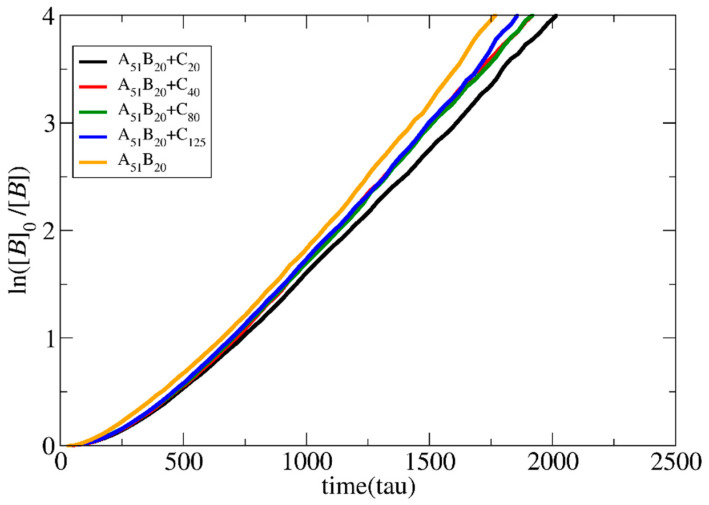
Pseudo-first-order kinetic plot of templated and non-templated polymerization for synthesis of A_51_B_20_ diblock copolymers for different template lengths: C_20_, C_40_, C_80,_ and C_125_. The total solution concentration is [*Φ*] = 0.24.

To study the effect of the template length on the polymerization rate, simulations of mixtures of 500 A_50_ neutral chains and a varying number (i.e., 500, 250, 125, and 80) of template chains consisting of 20, 40, 80, and 125 C-type beads, respectively, were performed at [*Φ*] = 0.24. The target length of the copolymer chains was set to A_51_B_20_. B–B and C–C interactions were kept attractive, corresponding to bad solvent conditions (T* = 2.0), while all other interactions were considered repulsive. As shown in Figure 7, the polymerization rate increases non-linearly with the increase in template length. In the mixtures with the longest template C_125_, the polymerization rate approaches the rate of non-templated polymerization, which is, in general, higher for [*Φ*] > 0.18. Long template chains may conform to an elongated shape, which favors the attractions with the oppositely charged monomers. This increases the monomer’s local concentration and the polymerization rate.

The effects of chemical feedback on the polymerization rate when the neutral chain length varies from 25 to 50 and 100 A-type beads for both templated and non-templated reactions were studied for the lowest concentration ([*Φ*] = 0.04). In all cases, the target length of the B-type block for the copolymers was fixed, i.e., A_26_B_20_, A_51_B_20,_ and A_101_B_20_. 

**Figure 8:**
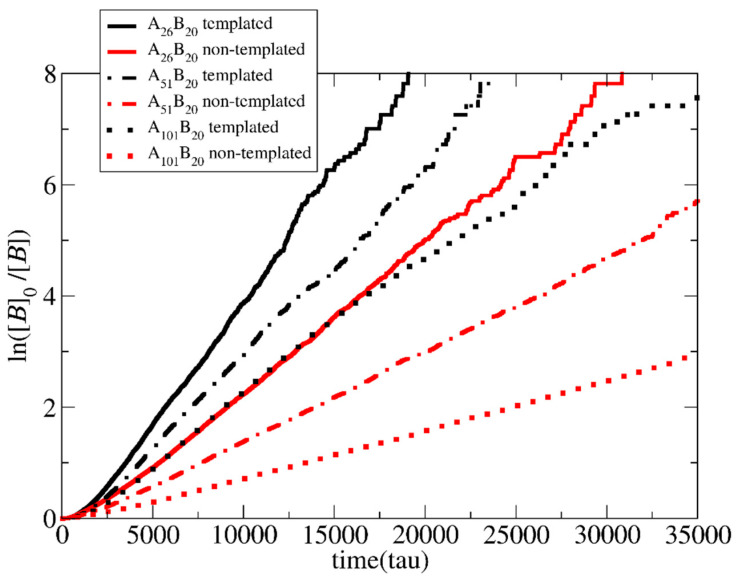
Pseudo-first-order kinetic plot of templated and non-templated polymerization for the synthesis of diblock copolymers with target length B_20_ from mixtures containing different neutral block chain lengths: A_25_, A_50_, and A_100_. The total solution concentration is [*Φ*] = 0.04. The template is a C_20_ chain.

As shown in Figure 8, in both the templated and the non-templated polymerization, the increase in the length of the neutral block leads to a linear decrease in the polymerization rate. This is because the excluded volume interactions between the A and B hinder monomers from approaching the active B-type end beads (reduced monomer concentration around the active centers). The decrease in the non-templated polymerization rate is enhanced compared to the templated polymerization. As discussed earlier, in the templated polymerization, a lot of monomers are stuck on the template, even before the polymerization starts. Thus, the excluded volume interactions with the A-type blocks concern a smaller number of free monomers. In contrast, in homogeneous non-templated polymerization, all monomers experience excluded volume interactions with A-type blocks. This hinders monomers from approaching the active end beads. 

### 3.2. Molecular Weights and Polydispersity

**Figure 9:**
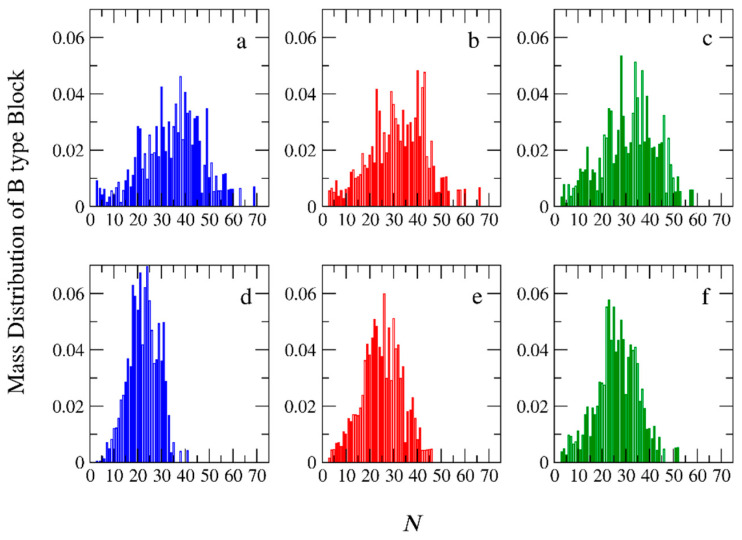
Mass distribution of the synthesized B-type copolymer block with target length of B_20_ for the templated polymerization of A_51_B_20_ copolymers at total solution concentrations ([*Φ*]) of (**a**) 0.04, (**b**) 0.12, (**c**) 0.24. (**d**–**f**) The mass distribution of B block for the non-templated polymerization for [*Φ*] = 0.04, 0.12, and 0.24 respectively. *N* is the B-type block molecular weight.

**Figure 10:**
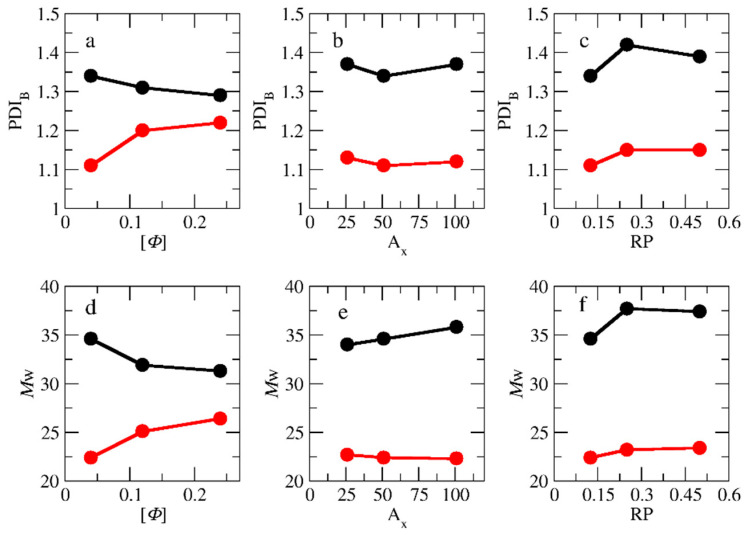
The variation of PDI_B_ and *M*w (**a**,**d**) with the total solution concentration ([*Φ*]) for templated (black) and non-templated (red circles) polymerization of A_51_B_20_ copolymers. (**b**,**e**) With the number of neutral block A-type beads and (**c**,**f**) with the reaction probability. The template is a C_20_ chain. [*Φ*] = 0.04.

The mass distributions of the B block of the A_51_B_20_ copolymers obtained from both types of polymerizations are presented in Figure 9 for [*Φ*] = 0.04, 0.12, and 0.24. A C_20_ template is used, and RP is set to 0.125. From these distributions, the number of (*M*n) and average molecular weight (*M*w), as well as the polydispersity index (PDI = *M*w/*M*n) can be calculated. The results are listed in Table S1 in the Supporting Information. From the simulations, we have verified that copolymer chains consisting of one or two B-type beads (i.e., A_51_B_1_, A_51_B_2_) do not participate in the micellization. Thus, these copolymers are considered impurities and are not counted in the calculation of *M*w, *M*n, and PDI. As can be seen in Table S1, the *M*n, *M*w, and PDI of the B-type block obtained from the templated polymerization are higher than the respective quantities in the non-templated polymerization, especially at low and moderate concentrations ([*Φ*] = 0.04 and 0.12). This is because, in the templated polymerization where half of the monomers are lying on the oppositely charged templates, many neutral chains remain without or with only 1 or 2 B-type beads. In the non-templated polymerization, where the monomers are homogeneously distributed in the solution, almost all A-type chains participate in the polymerization. In this case, the chains have narrow molecular weights and low PDI. Experimental results of the PDI values of the whole diblock copolymer chains (PDI_diblock_) and the pre-synthesized A-type precursors (PDI_B_) are reported [14]. To extract PDI_B_ and compare it with the simulation results, the following equation is used [33]:

(2) $${PDI}_{diblock}=w_{A}^{2}\left({PDI}_{A\;}-1\right)+w_{B}^{2}\left({PDI}_{B}-1\right)+1$$

where *w*_A_ and *w*_B_ are the ratios of the number of beads of the A and B block to the total number of beads in the diblock copolymer, respectively. Figure 10a,d show the PDI_B_ and *M*w as a function of [*Φ*], respectively. As can be observed, in the templated polymerization, the increase in the concentration decreases both the PDI_B_ and *M*w. In the non-templated homogeneous polymerization, the trend is the opposite; both PDI_B_ and *M*w increase with [*Φ*]. At [*Φ*] = 0.24, the difference between the PDI_B_ values for the two types of polymerizations becomes very small. Using the experimental values (${\mathrm{PDI}}_{\mathrm{diblock}}$ = 1.10 and ${\mathrm{PDI}}_{A}$ = 1.2) of Ding et al. [14], which are the same for both types of polymerizations at [*Φ*] = 0.5 in Equation (2), we predicted that the ${\mathrm{PDI}}_{B}$ = 1.2. This value is close to the simulation mean value of 1.26 at [*Φ*] = 0.24.

Our simulation results show that the difference in the PDI_B_ values between templated and non-templated polymerization at [*Φ*] = 0.04 is significant, which is in full agreement with the simulation results of Gavrilov et al. [15] for PISA polymerization. They found that the polydispersity of the diblock copolymer varies from 1.07 to 2.15 as the strength of the interactions between moieties is changed. However, no direct comparison with the experimental results can be performed because Bos et al. [13] do not report the PDI_B_ values for the non-templated polymerization. PDI and *M*w as functions of the A-type block length are presented in Figure 10b,e, respectively, for [*Φ*] = 0.04. It can be seen that the increase from A_26_ to A_51_ leads to a decrease in PDI. This is because the longer neutral chain covers the active end bead of the polymerized B block, preventing the monomers from approaching. The effect of RP on PDI and *M*w is presented in Figure 10c,f for [*Φ*] = 0.04. In the non-templated polymerization, the increase in the RP from 0.125 to 0.25 leads to an increase in *M*w and PDI values. However, a further increase to 0.5 has no extra effect on them because the local concentration of monomers around the active polymerization center cannot further increase. 

### 3.3. Micelle Size and Shape

**Figure 11:**
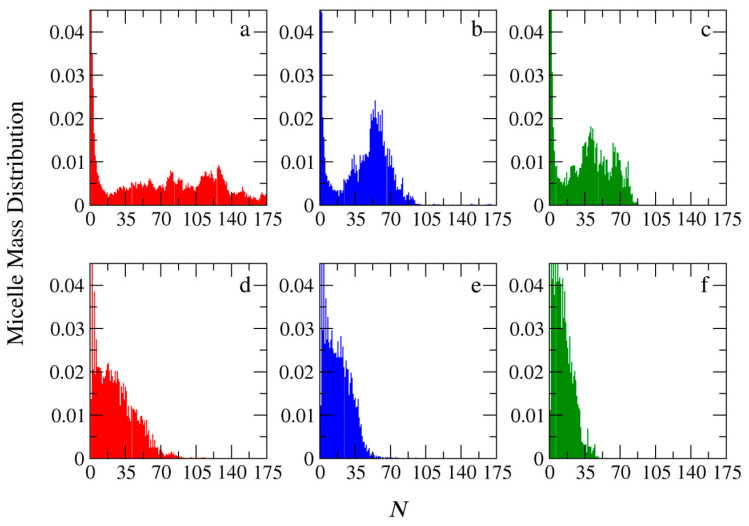
Mass distribution of micelles as a function of the aggregation number *N* formed by PIESA for the following systems: (**a**) A_26_B_20_ + C_20_, (**b**) A_51_B_20_ + C_20_, and (**c**) A_101_B_20_ + C_20_. Mass distribution of micelles formed during the two-step method for the following systems: (**d**) A_26_B_20_ + C_20_, (**e**) A_51_B_20_ + C_20,_ and (**f**) A_101_B_20_ + C_20_. In all simulations, [*Φ*] = 0.04.

The effects of the chemical feedback on the size and shape of the micelles were studied at the lowest concentrations, i.e., [*Φ*] = 0.04. The simulations of the PIESA one-step micellization were performed for the following mixtures: (a) A_25_ + C_20_, (b) A_50_ + C_20_, and (c) A_100_ + C_20_. The target diblock copolymers were A_26_B_20_, A_51_B_20_, and A_101_B_20_, respectively. To model the two-step micellization, after the end of polymerization, the C_20_ templates were added to the simulation box. The simulation scheme is described in detail in the model section. The mass distribution functions of the micelles computed from the molecular dynamics trajectories using the new Python code are shown in Figure 11 as a function of the aggregation number *N*.

Figure 11 clearly shows that in the templated polymerization, micelles with higher aggregation numbers are formed. Regardless of the micellization method, the increase of the neutral block in the diblock copolymers from A_26_B_20_ to A_51_B_20_ and further to A_101_B_20_ leads to smaller aggregates. This is expected because the increase in the hydrophilic block makes the corona of the micelle bulkier, better protecting the hydrophobic core formed by the complexation of the oppositely charged B and C beads. However, the mass distribution profiles obtained from the PIESA significantly differ from the two-step micellization. In PIESA, the mass distribution profile is a Gaussian-like function (micelles with preferential aggregate numbers are formed as shown in Figure 11b). The deviations from the Gaussian function Figure 11a,c can be attributed to the difficulties in the equilibration arising from the smaller neutral block and the higher *M*w of PIESA chains, respectively. In sharp contrast, the mass distribution profiles obtained from the two-step micellization do not reveal preferential aggregation [9] (decaying function with *N*, Figure 11d,e,f). The evolution of micelle mass distribution with the time in the templated polymerization is presented in Figure S2 for the mixture of A_51_B_20_ + C_20_. Initially, small micelles are formed; then, progressively, the aggregation number of micelles increases, which is in agreement with the TEM results presented in Figure S19 of reference [14]. After completion of the polymerization, the size rearrangements lead to the Gaussian-type distribution presented in Figure 11b. Snapshots of the simulation box are presented in Figure S3 for the same mixture at concentrations [*Φ*] = 0.04 and 0.36 and different simulation times (*τ*). It can be seen that the micelle size evolution with the progress of polymerization is in line with the TEM images presented in Ref. [14].

**Figure 12:**
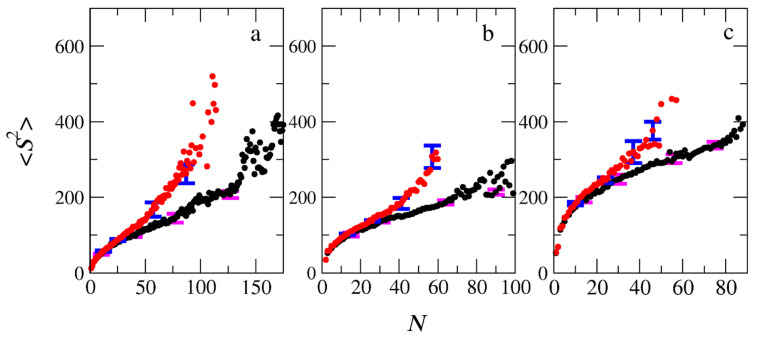
The mean squared radius of gyration of micelles formed by the PIESA (black circles) and by the two-step method (red circles) from mixtures (**a**) A_26_B_20_ + C_20_, (**b**) A_51_B_20_ + C_20,_ and (**c**) A_101_B_20_ + C_20_ as a function of the aggregation number *N*. Error bars represent standard deviation.

**Figure 13:**
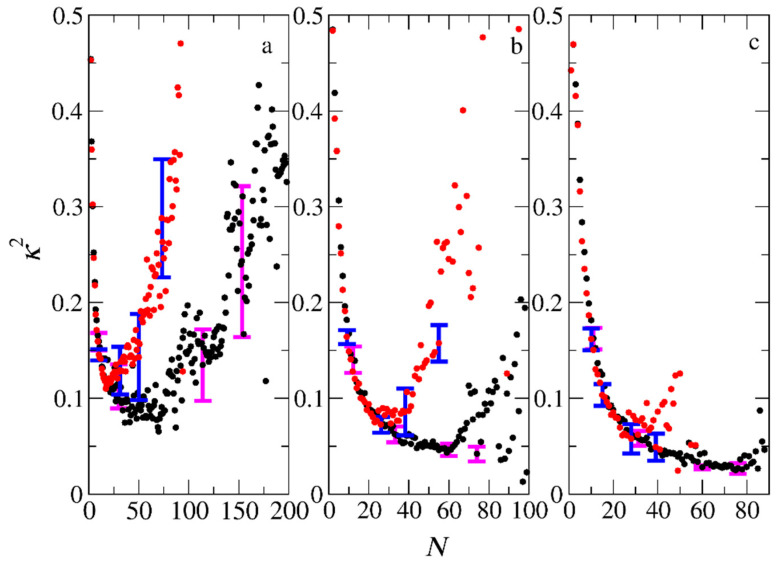
The mean shape anisotropy parameter *κ*^2^ of micelles formed by the PIESA (black circles) and by the two-step method (red circles) from mixtures (**a**) A_26_B_20_ + C_20_, (**b**) A_51_B_20_ + C_20,_ and (**c**) A_101_B_20_ + C_20_ as a function of the aggregation number *N*. Error bars represent standard deviation.

The mean squared radii of gyration (<*S*^2^>_PIESA_ and <*S*^2^>_two-step)_, describing the size of the micelles for the PIESA and the two-step micellization, respectively, are shown in Figure 12 as a function of the aggregation number. <*S*^2^>_two-step_ is always higher than <*S*^2^>_PIESA_. This finding is in line with the experimental findings of Bos et al. (Ref. [13], Figure 1d), where the two-step polymerization leads to very turbid samples and, consequently, to a higher size of aggregates than the templated polymerization. The deviation between <*S*^2^>_two-step_ and <*S*^2^>_PIESA_ decreases as the length of the neutral block forming the corona becomes much larger than the B-type block (Figure 12b,c). The lower values of <*S*^2^>_PIESA_ may be due to the higher PDI_B,_ which is 1.3 for PIESA, compared to 1.1 for the two-step micellization. Van der Kooij et al. [3] and Gavrilov et al. [15] have shown that the diblock copolymer chains with higher PDI resulted in denser packing in the micelle core. Thus, the overall mean squared radius of the gyration value of the polyelectrolyte complex micelles was much lower than the respective micelles formed by the low PDI copolymers. The shape anisotropy parameter [9,20] *κ*^2^ (Equation S2 in the Supporting Information) is shown in Figure 13 as a function of the aggregation number of the micelles for the two micellization schemes. From Figure 13, it is evident that micelles with very small aggregation numbers (*N* < 10) are elongated (*κ*^2^ > 0.1). The micelles are spherical (*κ*^2^ < 0.1) at moderate aggregation numbers, and again elongated for higher aggregation numbers (*N* > 90). The *κ*^2^ values for micelles with high aggregation numbers are scattered since this calculation suffers from bad statistics because such big micelles rarely form in the simulation. In general, micelles with aggregation numbers 20 < *N* < 80 formed by the PIESA micellization are more spherical than the ones in the two-step micellization for the A_26_B_20_ + C_20_ and A_51_B_20_ + C_20_ mixtures. In contrast, the micelles obtained from the A_101_B_20_ + C_20_ mixture (i.e., the system with the longest neutral A-type block) have similar shapes in both micellization schemes. The reason is that the shape of the large corona consisting of the A_101_ blocks predominantly determines the overall shape of the micelles.

**Figure 14:**
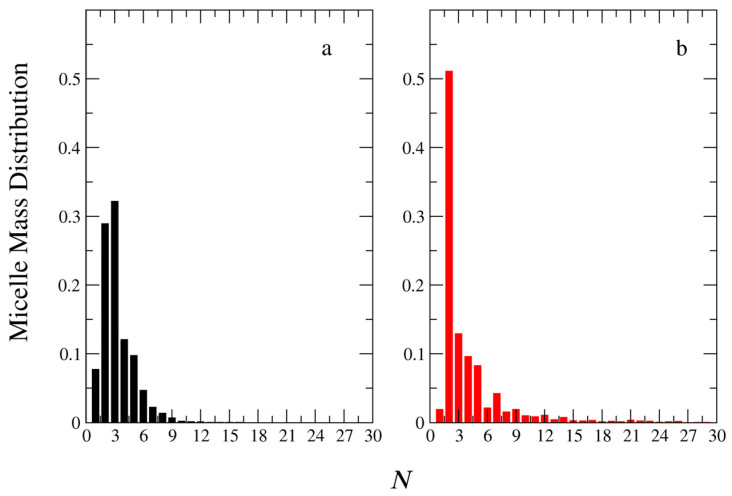
Mass distribution of micelles formed from mixtures of A_101_B_20_ + C_40_ as a function of the aggregation number (*N)* by (**a**) the PIESA method and (**b**) the two-step method. [*Φ*] = 0.04, RP = 0.125. The ratio of total negative to total positive charged beads is 2:1.

So far in this study, we have focused on micelles formed by mixtures in which the ratio of the charged template beads to the oppositely charged monomers is 1:1. To study the effect of chemical feedback in mixtures with an excess of templated negative beads, simulations were performed at a 2:1 ratio. The mass distributions of the micelles obtained from A_101_B_20_ + C_40_ mixtures are presented in Figure 14 as a function of the aggregation number for both PIESA and two-step schemes. Our results show that, regardless of the micellization method, only very small aggregates are formed (*N* ≤ 7). This finding is in full agreement with the experimental results of Boss et al. [13], and also agrees with previous theoretical predictions of PCMs being formed only at approximately equal charge stoichiometries [9].

## 4. Conclusions

To understand the effects of chemical feedback in the templated polymerization of a charged copolymer block and the simultaneous self-assembly with the oppositely charged template on the kinetics and the size of the resulting complex micelles, we performed CGMD simulations. The polymerization was based on a Monte Carlo stochastic reaction model. We show that chemical feedback fundamentally changes both the polymerization and self-assembly. At low solution concentrations ([*Φ*]), the location of monomers on the templates significantly accelerates the polymerization, while at high [*Φ*], the local monomer concentration on the templates becomes lower than that of the solution, thus slowing down the polymerization. This is in full agreement with the experimental data. Both the increase in the template length and the decrease in the neutral block length led to a linear increase in the polymerization rate.

In the templated polymerization, the *M*n, *M*w, and PDI values of the B-type block were computed to be higher than the respective quantities in the non-templated polymerization, especially at the low and moderate concentrations ([*Φ*] = 0.04 and 0.12). The micelles formed by the templated PIESA method have higher aggregation numbers than those formed by the two-step micellization. However, the <*S*^2^>_two-step_ is always higher than <*S*^2^>_PIESA_, which is in agreement with the experimental results. This is most probably a result of the higher PDI of the templated polymerization diblock copolymer block and the difference in the shape of micelles. The micelles with moderate aggregation numbers formed by the PIESA method are more spherical than the ones formed in the two-step micellization, which is in full agreement with experimental findings. This useful insight into the templated reaction assembly process in polymers obtained from molecular simulation is necessary for the rational design of new synthetic supramolecular materials. 

Here, we considered a simple polymerization scheme that included only initiators and propagation reactions without chain activation–deactivation and termination reactions since we mainly focused only on the effect of chemical feedback on the templated polymerization. For the study of other thermophysical properties, such as the phase diagrams of PIESA, the termination step through recombination is necessary to describe the complexity of the experimentally obtained phase diagrams [34].

## Data Availability

No data available.

## Supplementary Materials

The following supporting information can be downloaded at: https://www.mdpi.com/article/10.3390/polym15143024/s1: Autocorrelation function, shape anisotropy parameter, Effect of the reaction probability on polymerization rate and simulation box snapshots. Table S1: The Mn, Mw and PDI of the polymerized B type block for different total solution concentrations [*Φ*], reaction probabilities RP, lengths of template C type chains, lengths of neutral A type blocks, and polymerization methods; Table S2: The local monomer concentration in templated and non-templated reaction before the polymerization takes place (*τ* = 0) for different total solution concentration [*Φ*]. Figure S1: Pseudo-first-order kinetic plot of templated and non-templated polymerization for synthesis of A51B20 diblock copolymers for different reaction probabilities RP = 0.125, 0.25 and 0.5. The total solution concentration is [*Φ*] = 0.04; Figure S2: Mass distribution of micelles formed in the templated reaction assembly for the synthesis of A51B20 diblock copolymer calculated from single snapshot for different simulation time. (a) *τ* = 7500 (b) *τ* = 15,000, (c) *τ* = 22,500, (d) *τ* = 30,000. [*Φ*] = 0.04. The template is a C20 chain; Figure S3: Snapshots of the micelles formed in the templated reaction assembly for the synthesis of A51B20 diblock copolymer for different simulation time and concentration (a) *τ* = 6000, [*Φ*] = 0.04 (b) *τ* = 15,000, [*Φ*] = 0.04 (c) *τ* = 30,000, [*Φ*] = 0.04 (d) *τ* = 900, [*Φ*] = 0.36 (e) *τ* = 3000, [*Φ*] = 0.36 (f) *τ* = 30,000, [*Φ*] = 0.36 The template is a C20 chain. The micelle size evolution is in line with the TEM images presented in ref. [14]. References [9,19,20,35] are cited in the supplementary materials.
