# Supplementary material for: Chemical Feedback in Templated Reaction-Assembly of Polyelectrolyte Complex Micelles: A Molecular Simulation Study of the Kinetics and Clustering
Source: Polymers (Basel). 2023 Jul 12;15(14):3024. doi: 10.3390/polym15143024 (PMC10383549; doi:10.3390/polym15143024)
Supplement: Supplementary file 1 [file polymers-15-03024-s001.zip › polymers-2466521-supplementary.pdf]

Supporting Information

Chemical Feedback in Templated Reaction-Assembly of Polyelectrolyte Complex Micelles: A Molecular Simulation Study of the Kinetics and Clustering

Christos Gioldasis[1], Apostolos Gkamas[1*], Othonas Moultos[2] and Costas Vlahos[1*]

[1] Chemistry Department, University of Ioannina, 45110 Ioannina, Greece

[2] Process& Energy Department, Delft University of Technology, Delft, The Netherlands

Table S1 The *M*n, *M*w and PDI of the polymerized B type block for different total solution concentrations [Φ], reaction probabilities RP, lengths of template C type chains, lengths of neutral A type blocks, and polymerization methods.

	M_n (B-block)	M_w (B-block)	PDI_B
**Templated polymerization**			
$A_{51}B_{20}+C_{20}$ [Φ]=0.04, RP=0.125	25.9	34.6	1.34
$A_{51}B_{20}+C_{20}$ [Φ]=0.12, RP=0.125	24.4	31.9	1.31
$A_{51}B_{20}+C_{20}$ [Φ]=0.24, RP=0.125	24.2	31.3	1.29
$A_{51}B_{20}+C_{80}$ [Φ]=0.24, RP=0.125	23.6	30.2	1.28
$A_{26}B_{20}+C_{20}$ [Φ]=0.04 , RP=0.125	24.8	34.0	1.37
$A_{101}B_{20}+C_{20}$ [Φ]=0.04, RP=0.125	26.1	35.8	1.37
$A_{51}B_{20}+C_{20}$ [Φ]=0.04, RP=0.25	26.6	37.7	1.42
$A_{51}B_{20}+C_{20}$ [Φ]=0.04, RP=0.5	26.8	37.4	1.39
$A_{51}B_{40}+C_{40}$ [Φ]=0.04, RP=0.5	47.1	68.8	1.46
$A_{101}B_{40}+C_{40}$ [Φ]=0.04, RP=0.125	49.5	71.9	1.45
**Non-templated polymerization**			
$A_{51}B_{20}$ [Φ]=0.04, RP=0.125	20.2	22.4	1.11
$A_{51}B_{20}$ [Φ]=0.12, RP=0.125	21.0	25.1	1.20
$A_{51}B_{20}$ [Φ]=0.24, RP=0.125	21.6	26.4	1.22
$A_{26}B_{20}$ [Φ]=0.04, RP=0.125	20.1	22.7	1.13
$A_{101}B_{20}$ [Φ]=0.04, RP=0.125	19.9	22.3	1.12
$A_{51}B_{20}$ [Φ]=0.04, RP=0.25	20.2	23.2	1.15
$A_{51}B_{20}$ [Φ]=0.04, RP=0.5	20.3	23.4	1.15
$A_{51}B_{40}$ [Φ]=0.04, RP=0.125	40.0	45.6	1.14
$A_{101}B_{40}$ [Φ]=0.04, RP=0.125	39.9	45.5	1.14

Autocorrelation Function

The duration of the simulation was evaluated by calculating the tracer autocorrelation function [9,19]:

$$C(t) = \frac{\langle N(t_0+t)N(t_0)\rangle - \langle N(t_0)\rangle^2}{\langle N^2(t_0)\rangle - \langle N(t_0)\rangle^2} \quad , \qquad \text{(S1)}$$

where $N(t)$ is the number of molecules in the micelle in which the copolymer resides at time t. We took all copolymers as tracers, and every time step as a time origin t_0. The characteristic relaxation time t_{relax} is defined as the required time for $C(t)$ to reach the value [19] of 1/e = 0.37.

Shape anisotropy parameter

The shape anisotropy κ^2 is defined as [9,20,35]:

$$\kappa^2 = 1 - 3\frac{<I_2>}{<I_1{}^2>} \ , \qquad \text{(S2)}$$

where I_1 and I_2 are the first and second invariants of the radius of gyration tensor. $\kappa^2 = 0$ corresponds to a perfect sphere while $\kappa^2 = 1$ to a perfect rod.

Effect of the reaction probability value on the polymerization rates

In all simulations discussed thus far, the total polymerization reaction probability was set to RP=0.125. To investigate the sensitivity of the computed templated and non-templated

polymerization rates for differed RP, simulations of mixtures of A_{50} chains with B type monomers at [Φ]=0.04 were carried out for RP= 0.125, 0.25 and 0.5. The target length of the synthesized diblock copolymer was set to $A_{51}B_{20}$. The obtained kinetic diagrams are presented in **Figure S1**. As expected, the increase in RP from 0.125 to 0.25 results in the increase of both polymerization rates. In the case of non- templated polymerization, further increase of RP from 0.25 to 0.5 leads to a smaller increase of the reaction rate than that of the templated polymerization. This is because the monomer concentration, around the template, is higher than the concentration of monomers in the homogeneous non-templated polymerization at [Φ]=0.04.

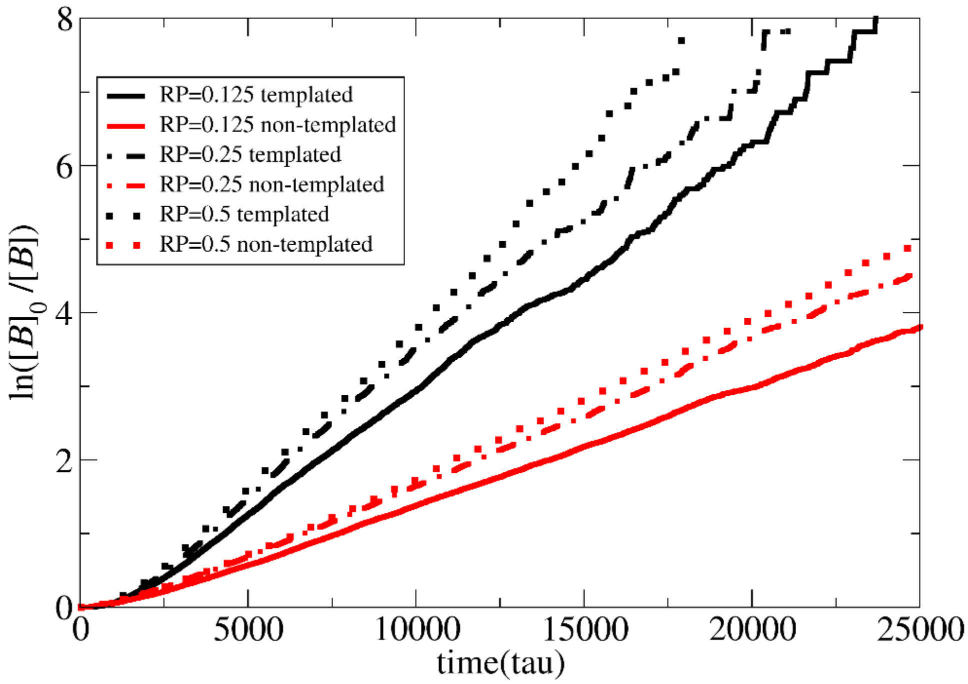

Figure S1 Pseudo-first-order kinetic plot of templated and non-templated polymerization for synthesis of $A_{51}B_{20}$ diblock copolymers for different reaction probabilities RP= 0.125, 0.25 and 0.5. The total solution concentration is [Φ]=0.04.

**[Φ]**	**Local concentration in templated polymerization**	**Local concentration in non-templated polymerization**	**Ratio of local concentrations**
**0.04**	0.0158	0.0086	1.837
**0.12**	0.0289	0.0258	1.120
**0.24**	0.0483	0.0516	0.936
**0.36**	0.0689	0.0774	0.890

Table S2 The local monomer concentration in templated and non-templated reaction before the polymerization takes place (τ =0) for different total solution concentration [Φ].

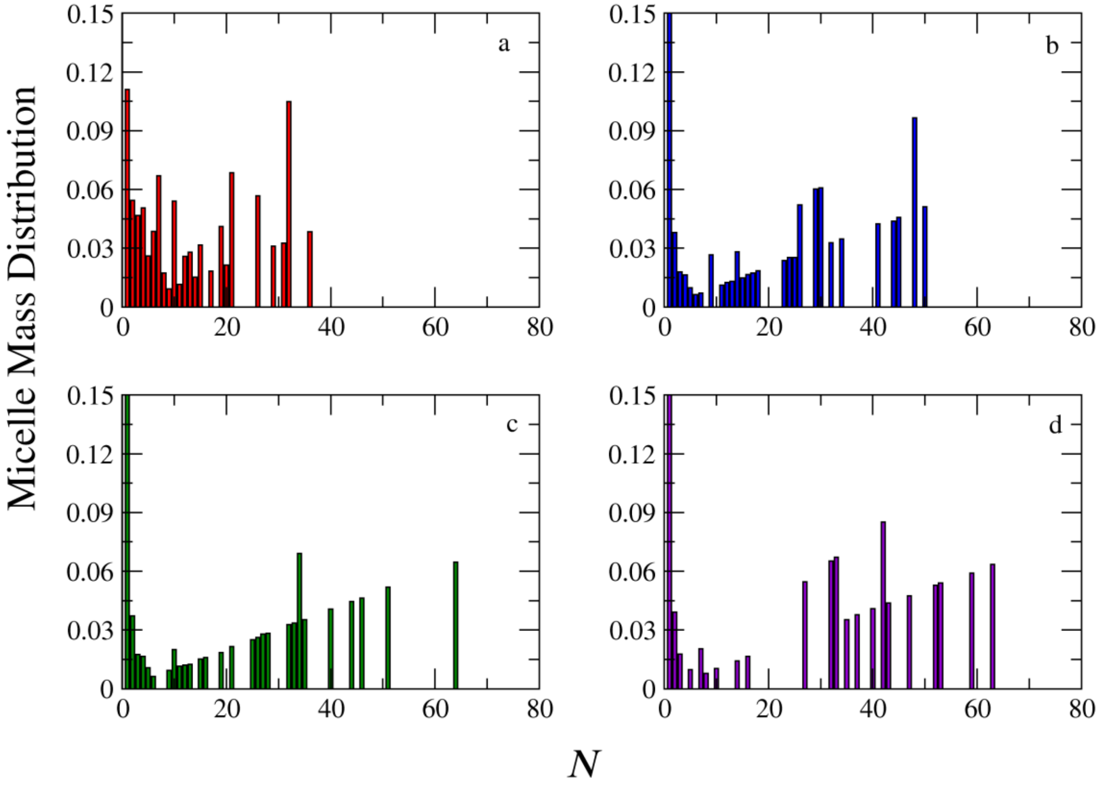

Figure S2 Mass distribution of micelles formed in the templated reaction assembly for the synthesis of $A_{51}B_{20}$ diblock copolymer calculated from single snapshot for different simulation time. (a) τ = 7500 (b) τ = 15000, (c) τ = 22500, (d) τ = 30000. [Φ]=0.04. The template is a C_{20} chain.

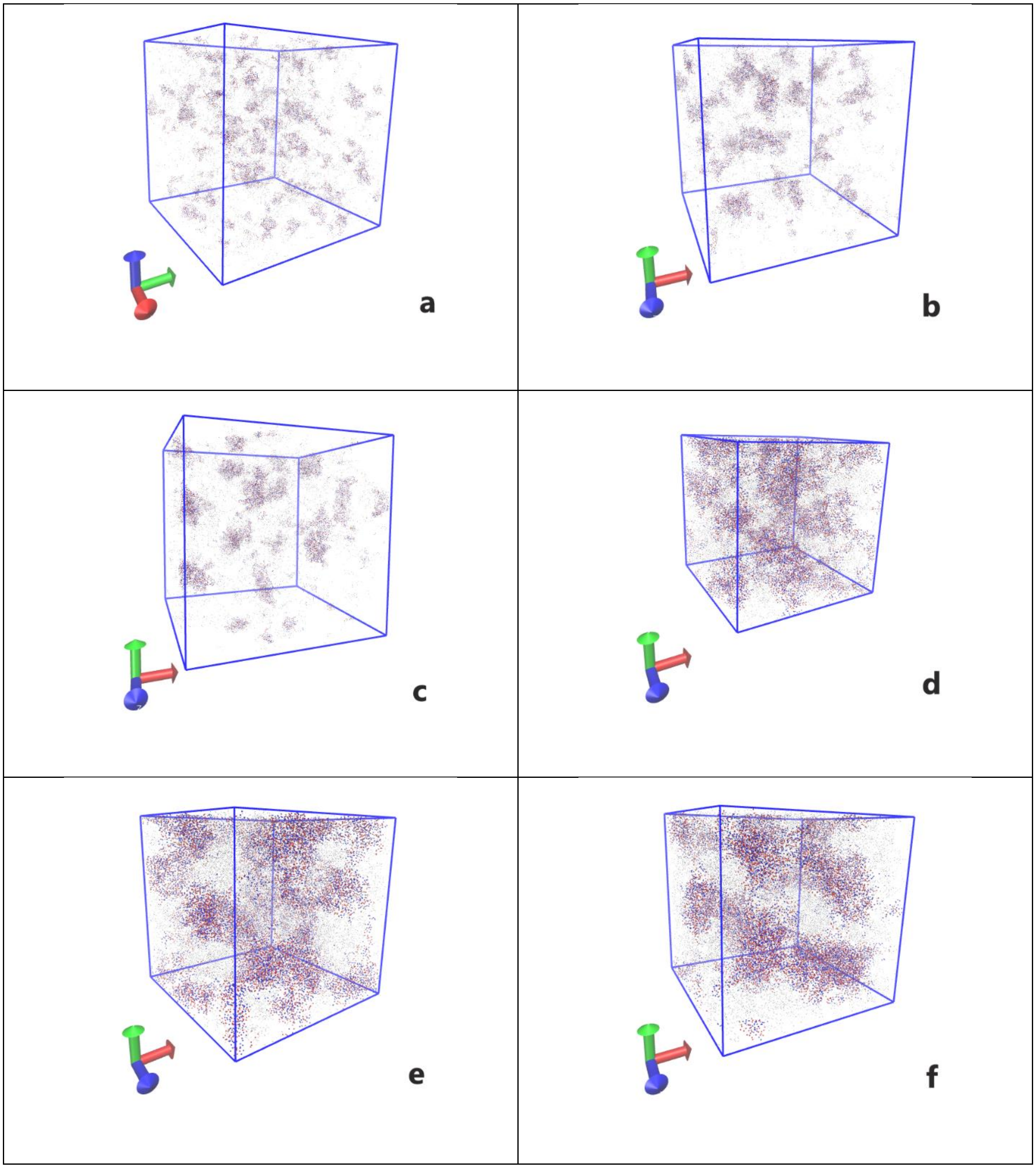

Figure S3 Snapshots of the micelles formed in the templated reaction assembly for the synthesis of $A_{51}B_{20}$ diblock copolymer for different simulation time and concentration (a) τ = 6000, [Φ]=0.04 (b) τ = 15000, [Φ]=0.04 (c) τ = 30000, [Φ]=0.04 (d) τ = 900, [Φ]=0.36 (e) τ = 3000, [Φ]=

0.36 (f) τ = 30000, [Φ]= 0.36 The template is a C_{20} chain. The micelle size evolution is in line with the TEM images presented in ref. [14].

1. Gioldasis, C.; Gergidis, L.N.; Vlahos, C. Micellization through complexation of oppositely charged diblock copolymers: Effects of composition, polymer architecture, salt of different valency, and thermoresponsive block. *J. Polym. Sci.* **2021**, *59*, 191–204. https://doi.org/10.1002/pol.20200754.
2. Suek, N.W.; Lamm, M.H. Computer Simulation of Architectural and Molecular Weight Effects on the Assembly of Amphiphilic Linear−Dendritic Block Copolymers in Solution. *Langmuir* **2008**, *24*, 3030–3036. https://doi.org/10.1021/la703006w.
3. Miliou, K.; Gergidis, L.N.; Vlahos, C. Polyelectrolyte micelles in salt-free solutions: Micelle size and electrostatic potential. *J. Polym. Sci. Part B Polym. Phys.* **2018**, *56*, 924–934. https://doi.org/10.1002/polb.24608.
4. Theodorou, D. N.; Suter, U. W. Shape of Unperturbed Linear Polymers: Polypropylene. *Macromolecules* **1985**, 18 (6), 1206–1214. https://doi.org/10.1021/ma00148a028.
